# Complex PTSD symptom clusters and executive function in UK Armed Forces veterans: a cross-sectional study

**DOI:** 10.1186/s40359-024-01713-w

**Published:** 2024-04-15

**Authors:** Natasha Biscoe, Emma New, Dominic Murphy

**Affiliations:** 1 Combat Stress, Leatherhead, Surrey KT22 0BX UK; 2 Birmingham and Solihull Mental Health NHS Foundation Trust, Birmingham, UK; 3 King’s Centre for Military Health Research, King’s College London, London, SE5 9PR UK

**Keywords:** Veterans, Mental health, PTSD, Complex PTSD, Executive function, Emotion dysregulation

## Abstract

**Background:**

Less is known about complex posttraumatic stress disorder (CPTSD) than postrraumatic stress disorder (PTSD) in military veterans, yet this population may be at greater risk of the former diagnosis. Executive function impairment has been linked to PTSD treatment outcomes. The current study therefore aimed to explore possible associations between each CPTSD symptom cluster and executive function to understand if similar treatment trajectories might be observed with the disorder.

**Methods:**

A total of 428 veterans from a national charity responded to a self-report questionnaire which measured CPTSD symptom clusters using the International Trauma Questionnaire, and executive function using the Adult Executive Function Inventory. Single and multiple linear regression models were used to analyse the relationship between CPTSD symptom clusters and executive function, including working memory and inhibition.

**Results:**

Each CPTSD symptom cluster was significantly associated with higher executive function impairment, even after controlling for possible mental health confounding variables. Emotion dysregulation was the CPTSD symptom cluster most strongly associated with executive function impairment.

**Conclusions:**

This is the first study to explore the relationship between executive function and CPTSD symptom clusters. The study builds on previous findings and suggests that executive function could be relevant to CPTSD treatment trajectories, as is the case with PTSD alone. Future research should further explore such clinical implications.

## Background

Military veterans face a greater risk of experiencing PTSD than the general UK population [1] and are more likely to meet criteria for Complex PTSD (CPTSD) than PTSD [2]. PTSD encompasses a set of symptoms which may be experienced following a traumatic event, including hyperarousal, re-experiencing (nightmares, intrusions), cognitive and behavioural avoidance and negative alterations in mood (DSM-V; [3]). CPTSD was added to the International Classification of Diseases in 2011 [4] as a distinct disorder. A diagnosis of CPTSD includes experiencing clusters of symptoms that encompass PTSD, as well as symptom clusters referred to as Disturbances in Self-Organisation (DSO), which are: emotion dysregulation, interpersonal difficulties, and negative self-concept, as well as functional impairment connected to both PTSD and DSO symptoms.

CPTSD has been linked with sustained and multiple traumas [5] as well as interpersonal trauma [6]. Military veterans appear to be at greater risk of CPTSD than PTSD [7]. Indeed, CPTSD appears to be more prevalent in UK treatment-seeking veterans than PTSD (with 80% meeting criteria for CPTSD compared to 20% for PTSD; [2, 8]. Additionally, proportionally higher treatment dropout rates are reported for veterans with CPTSD [9]. It is therefore clinically important to understand factors which may be relevant to both PTSD and CPTSD, as interventions may need to be tailored to each disorder respectively.

### PTSD and executive function

An association between impairments in executive function (EF), and posttraumatic stress disorder (PTSD) is well-established in the literature (for review see: [10–12]). EFs are a collection of abilities grouped together for their relevance to planning and executing complex, goal-directed behaviour [13–15]. There is significant variation in both definitions of the concept and how the construct is operationalised, although the current study follows Miyake and colleagues [16] as this conceptualisation aligns well with the self-report measure of executive function used in this study. These authors identify cognitive flexibility, working memory and inhibition as core EFs, deficits in all of which may be relevant to PTSD [17–21]. Furthermore, one study has reported that greater inhibitory control is associated with a better improvement in PTSD symptoms following psychological treatment, indicating the possible relevance of EF in PTSD recovery trajectories [22]. Less is known about whether similar trajectories would be observed in those with CPTSD. However, insight may be drawn from neurocognitive explanations of the observed associations between EF and PTSD.

### Neurocognitive models of PTSD and EF

Several meta-analyses of lesion and neuroimaging studies implicate the prefrontal cortex (PFC) as key in supporting EF [23–25]. The PFC has been theorised as a control centre, mediating between sensory inputs and behavioural outputs via regulation of brain systems central to emotion processing such as the amygdala [26]. The PFC is also structurally associated with PTSD, as well as the amygdala, hippocampus, and cingulate cortex [27], with this system key to attaching emotional valence to memories relevant to the fear-based experiences that lead to PTSD [19].

The shared relevance of these brain systems to both EF and PTSD suggests a neurocognitive explanation for the overlap observed between the two constructs. For example, one neurocognitive model of PTSD posits that PFC (and associated deficits in EF) may be ineffectively regulating hyperarousal of the amygdala in individuals with PTSD when a perceive threat is observed in a safe environment [28–30]. Furthermore, elevated arousal – a symptom of PTSD – may deplete cognitive resources leading to deficits in EF as attention is focused instead on regulating hyperarousal [20, 31–33].

### EFs and CPTSD

Neuroimaging studies reinforce this theory and suggest functional connectivity between the PFC and brain regions relevant to emotion regulation are key to supporting EF [34, 35]. Emotion dysregulation therefore may be pertinent to the observed overlap between PTSD and EF. Given emotion dysregulation is a DSO symptom of CPTSD, exploring associations between CPTSD and EFs could inform understanding of the disorder and how existing PTSD interventions could be tailored to improve treatment response in veterans seeking treatment for CPTSD. In a study using an adolescent sample, deficits in EFs were associated with greater CPTSD severity [36]. However, less is known about the relationship between CPTSD and EFs in veteran populations.

### The current study

Given the potential relevance of EF to PTSD treatment outcomes in veterans, and the need to further understand CPTSD in this population, the current study explores the relationship between both PTSD and CPTSD and a self-report measure of EF (inhibition and working memory) in a clinical sample of UK veterans. Associations between each PTSD symptom cluster and EFs are separately investigated, including the DSO clusters that encompass CPTSD. In line with previous studies [36], it is hypothesised that lower executive functioning scores (both working memory and inhibition) will be associated with greater severity of CPTSD symptoms.

## Methods

### Ethics

This study was approved by [blinded for review].

### Participants

Of the veterans seeking treatment UK charity, a 20% random sample was selected to assess whether they met study inclusion criteria: (1) having a valid email address; (2) having provided consent to contact from the research team about studies; (3) had attended one or more appointments (classed as treatment-seeking). In total 989 veterans were emailed with the study link, to which 428/989 responded (43.3% response rate; M_age_=50.4, SD_age_=10.9). Participation was voluntary. No differences were found between those who returned completed questionnaires and non-responders [2]. We determined this by analysing predictors of returning a completed survey, including age, sex and service branch.

### Procedure

Eligible and consenting veterans were emailed the link to a self-report questionnaire hosted on Survey Monkey, which included demographic questions and the measures described below. Responses were collected between August and October 2020 and participants were emailed not more than five times. The questionnaire took approximately 20 min to complete. Full study procedure has been described previously [2].

### Measures

#### ADEXI

The Adult Executive Function Inventory (ADEXI; [37]), measures EF on a 14-item self-report scale, with responses on a five-point Likert scale ranging from zero (definitely not true) to four (definitely true). Items 1, 2, 5, 7, 8, 9, 11, 12 and 13 comprise the working memory subscale, e.g.: “I have difficulty remembering lengthy instructions” and “when someone asks me to do several things, I sometimes only remember the first or last”. The remaining items make up the inhibition subscale, e.g.: “I have a tendency to do things without first thinking about what could happen” and “I sometimes have difficulty stopping myself from doing something that I like even though someone tells me that it is not allowed”. A higher score on the scale or each of the subscales indicates greater impairment. The ADEXI has good internal consistency and test-retest reliability, but poor convergent validity with neuropsychological tests of EF [37]. The ADEXI has good internal consistency (α = 0.68–0.72; [37]).

#### *ITQ*

Symptoms of PTSD and CPTSD were measured using the International Trauma Questionnaire [38], an 18-item scale with responses on a 5-point Likert scale ranging from zero (not at all) to four (extremely). Two items measure each of the three PTSD symptom clusters: hyperarousal, re-experiencing and avoidance. Two items measure each of the three disturbances in self-organisation (DSO) symptom clusters that comprise CPTSD: negative self-concept, interpersonal relationships and affect dysregulation. Three identical items then measure functional impairment related to the PTSD and DSO symptom clusters respectively. The ITQ has strong psychometric properties [39]. Possible caseness for PTSD is indicated by a score of two or higher on at least one of each item measuring each PTSD symptom cluster, as well scoring two or higher on one of the three functional impairment items relating to PTSD symptom clusters. Possible caseness for CPTSD is indicated by meeting the criteria for PTSD, as well as scoring two or higher on at least one of the two items for each DSO symptom cluster, and at least a two on one of the functional impairment items relating to DSO symptoms. The ITQ has good internal consistency (α = 0.90; [39]).

#### *GHQ-12*

Symptoms of generalised anxiety and depression were measured with the General Health Questionnaire (GHQ-12; [40]), a 12-item scale where a score of four or higher is indicative of potential caseness for common mental health difficulties (CMDs). The GHQ-9 has good internal consistency (α = 0.72; [41]).

#### *PHQ-15*

Somatic symptoms were measured using the Patient Health Questionnaire (PHQ-15; [41]), a 15-item scale where a score above 15 indicates higher severity of somatic symptoms. The PHQ-15 has good internal consistency (α = 0.80; [42]).

#### *SCI*

Symptoms of poor sleep quality were measured using the Sleep Condition Indicator (SCI; [43]), an eight-item scale where a score below 16 is indicative of a potential insomnia disorder. The SCI has good internal consistency (α = 0.86; [44]).

#### *DAR-5*

Symptoms of difficulties with anger were measured using the Dimensions of Anger Reactions (DAR-5; [45]), a five-item scale where a score higher than 12 is indicative of possible anger difficulties. The DAR-5 has good internal consistency (α = 0.89–0.90; [46]).

#### AUDIT

Symptoms of alcohol misuse were measured using the Alcohol Use Disorders Identification Test (AUDIT; [47]), a 10-item scale where scores higher than eight and 16 respectively are classified as possible hazardous and harmful alcohol use. The AUDIT has good internal consistency (α = 0.60–0.80; [48]).

### Data analysis

Data were prepared in STATA 13.0 and analysed in SPSS v.26. Continuous variables were ADEXI scores and subscale scores. These were averaged so that comparisons could be made across scores calculated from different numbers of items. All other variables were categorical, divided into case and no case or high severity and lower severity for each health outcome, and no PTSD, PTSD, and CPTSD for the ITQ variable. To understand the relationship between mental health variables, including PTSD and EF, single linear regression models were used with demographic and mental health caseness variables as predictors, and ADEXI and inhibition and working memory subscale scores as outcome variables in separate analyses. This was to understand possible confounding variables for any relationship between PTSD and CPTSD with EF. Multiple linear regression models were then used with PTSD and CPTSD caseness as predictor variables, and ADEXI score, and subscale scores as outcome variables. Those variables which were significant in the single linear regression models were included in the multiple regression models to adjust for possible confounding factors. Single linear regression models explored the relationships between individual PTSD and DSO symptom clusters with EF. ‘Caseness’ for each symptom cluster was calculated as a score of two or higher on at least one of the two items measuring each cluster. The sample met assumptions for multiple linear regression: the data were normally distributed (W = 0.96, *p* = 0.23), there was low multicollinearity and there is a linear relationship between the variables used in the regression models. As described in [2], analyses were restricted to responders only and missing data were not included in the models due to the assumption that data were missing at random. A power analysis was not conducted for the present study as the analysis was exploratory and data were collected through convenience sampling [49]. In regression analysis, B values below 0.1. between 0.1 and 0.5 and above 0.5 are broadly considered small, medium and high respectively [50].

## Results

Demographic characteristics are described in Table 1, as well as descriptive statistics for the variables included in regression models.

**Table 1:** Demographic characteristics of the full sample

	Full sample M (SD)
Age (years)	50.5 (10.9)
**Gender**	n (%)*
Male	417 (97.4)
Female	11 (2.6)
**Ethnicity**	
White	379 (94.8)
Ethnic minority	21 (5.2)
**Relationship status**	
Single, divorced, separated, widowed	266 (66)
In a relationship	137 (34)
**Employment status**	
Working or retired	223 (56.3)
Not working	173 (43.7)
**Housing status**	
No fixed address	36 (9)
Fixed address	367 (91)

### Single regression models

Single linear regression models for demographic and mental health factors are presented in Table 2. Being unemployed and having an ethnicity other than white were significantly associated with higher overall EF, inhibition and working memory impairment. Having high somatic symptoms and meeting caseness for probable common mental health difficulties were also associated with higher overall EF, inhibition and working memory impairment. In addition, scores indicating hazardous alcohol use were associated with working memory and inhibition impairment, and sleep disturbances were associated with a higher working memory impairment.

**Table 2:** Executive functioning, working memory, and inhibition descriptive statistics by health outcome

	Executive Functioning total (ADEXI)	Working memory	Inhibition
PTSD Group	M (SD)		
No PTSD	2.76 (0.76)	2.70 (0.87)	2.88 (0.78)
PTSD	2.97 (0.65)	2.98 (0.78)	2.95 (0.70)
CPTSD	3.39 (0.72)	3.42 (0.84)	3.34 (0.78)
**Hazardous drinking (AUDIT)**			
Case	3.23 (0.76)	3.18 (0.89)	3.07 (0.83)
No case	3.11 (0.79)	3.10 (0.95)	3.02 (0.75)
**Sleeping difficulties (SCI)**			
Case	3.23 (0.79)	3.24 (0.90)	3.21 (0.82)
No case	3.06 (0.76)	3.03 (0.93)	3.10 (0.77)
**Common mental health difficulties (GHQ-12)**			
Case	3.26 (0.75)	3.27 (0.88)	3.23 (0.78)
No case	2.76 (0.82)	2.71 (0.93)	2.90 (0.84)
**Somatisation (PHQ-15)**			
Case	3.55 (0.71)	3.61 (0.83)	3.46 (0.78)
No case	2.97 (0.75)	2.94 (0.86)	3.02 (0.78)

PTSD = posttraumatic stress disorder; CPTSD = complex posttraumatic stress disorder. AUDIT = Alcohol Use Disorder Identification Test. SCI = Sleep Condition Indicator. GHQ = General Health Questionnaire. PHQ = Patient Health Questionnaire

### Multiple regression models

Multiple regression models for PTSD adjusted for all other significant variables besides CPTSD caseness observed in the single regression models. The same models were analysed including CPTSD as a predictor and not PTSD caseness. These models are displayed in Table 3. Across all adjusted models, both PTSD and CPTSD remained significant predictors for EF, inhibition and working memory.

**Table 3:** Single linear regression models for demographic and health variables and executive functioning scores

	Executive function Adjusted B (95% CI)	Working memory Adjusted B (95% CI)	Inhibition B (95% CI)
Age	-0.01 (-0.01-0.01)	-0.01 (-0.01-0.00)	-0.01 (-0.01-0.00)
Gender	0.05 (-0.45-0.54)	-0.18 (-0.75-0.39)	0.42 (-0.06-0.91)
Ethnicity	0.62 (0.27–0.97)**	0.71 (0.30–1.12)**	0.46 (0.10–0.82)**
Relationship status	0.01 (-0.12-0.23)	0.06 (-0.14-0.26)	0.06 (-0.12-0.23)
Employment status	0.19 (0.03–0.35)**	0.20 (0.12–0.39)**	0.17 (0.01–0.33)**
Level of education	0.13 (-0.03-0.30)	0.12 (-0.07-0.31)	0.16 (-0.01-0.33)
PTSD	0.60 (0.43–0.76)**	0.69 (0.50–0.88)**	0.43 (0.25–0.60)**
CPTSD	0.59 (0.44–0.75)**	0.68 (0.50–0.86)**	0.45 (0.28–0.62)**
CMDs (GHQ-12)	0.49 (0.29–0.70)**	0.54 (0.30–0.78)**	0.33 (0.11–0.54)**
Somatisation (PHQ-15)	0.58 (0.43–0.74)**	0.67 (0.49–0.85)**	0.44 (0.28–0.61)**
Hazardous drinking (AUDIT)	0.13 (-0.05-0.31)	0.07 (-0.14-0.28)	0.24 (0.06–0.42)**
Sleep difficulties (SCI)	0.17 (-0.01-0.34)	0.21 (0.01–0.41)**	0.11 (-0.07-0.29)

No case is the reference category in each linear regression model. ADEXI and subscale scores are the outcome in each linear regression model. ** = *p* < 0.05. PTSD = posttraumatic stress disorder; CPTSD = complex posttraumatic stress disorder. AUDIT = Alcohol Use Disorder Identification Test. SCI = Sleep Condition Indicator. GHQ = General Health Questionnaire. PHQ = Patient Health Questionnaire

### PTSD and DSO symptom clusters

Linear regression models for each of the PTSD and DSO symptom clusters and EF, inhibition and working memory are displayed in Table 4. In line with our hypothesis, each symptom cluster was significantly associated with EF, as well as inhibition and working memory subscales.

**Table 4:** Linear regression models for PTSD and CPTSD symptom clusters

	Executive function B (95% CI)	Working memory B (95% CI)	Inhibition B (95% CI)
**PTSD symptoms**			
Re-experiencing	0.14 (0.10–0.17)**	0.16 (0.13–0.20)**	0.08 (0.05–0.12)**
Avoidance	0.13 (0.10–0.16)**	0.15 (0.11–0.19)**	0.10 (0.06–0.13)**
Current threat	0.15 (0.12–0.19)**	0.17 (0.13–0.21)**	0.12 (0.08–0.15)**
**DSO symptoms**			
Affect dysregulation	0.18 (0.14–0.22)**	0.20 (0.15–0.24)**	0.15 (0.11–0.19)**
Negative self-concept	0.14 (0.11–0.17)**	0.16 (0.12–0.19)**	0.11 (0.07–0.14)**
Relationship disturbances	0.15 (0.12–0.18)**	0.16 (0.13–0.20)**	0.12 (0.09–0.15)**

A score of > 2 on at least one of two ITQ items for each symptom cluster indicates symptom presence. Not having the symptom is the reference category in each linear regression model. ADEXI and subscale scores are the outcome in each model. ** = *p* < 0.05. PTSD = posttraumatic stress disorder. DSO = disturbances in self-organisation

## Discussion

The aim of the current study was to explore the associations between CPTSD symptom clusters and EF in a clinical sample of UK veterans. Both PTSD and CPTSD caseness were significantly associated with greater impairment in inhibition and working memory, in line with our hypothesis. All PTSD symptom clusters, and the DSO symptom clusters which encompass CPTSD, were associated with inhibition and working memory. In particular, the DSO symptom emotion dysregulation was most strongly associated with EF impairment. PTSD encompasses symptoms hyperarousal, re-experiencing and avoidance. CPTSD is a relatively new separate diagnosis which includes PTSD symptoms as well as DSO symptoms: emotion dysregulation, negative self-concept and interpersonal difficulties, as well as functional impairment relating to these domains [4].

These associations remained after controlling for the following possible confounders, which were also found to be associated with greater EF impairment: employment status, ethnicity, somatisation severity, common mental health disorders, alcohol misuse and for working memory, sleep function. The finding that EF impairment is associated with worse health coheres with previous research, which has observed relationships between EF deficits and both depression [51] and somatisation disorder [52]. Additionally, sleep deprivation is consistently associated with impairments in working memory [53, 54].

### Emotion dysregulation and EF impairment

Our finding that emotion dysregulation was the CPTSD symptom cluster most associated with EF coheres with and builds on neurocognitive models espoused in the literature. Previous research has suggested functional connectivity between the PFC and limbic system is key in the overlap observed between PTSD chronicity, severity, and EF impairment [10, 55]. In one study, those with greater functional connectivity in this system - termed the frontal parietal control and limbic network (FPCN) - were observed to have less chronicity of and greater reduction in PTSD symptoms [56]. The FPCN underlies emotion processing [57], mind wandering [58] and is neurally connected with the default mode network (DMN; [59]), all of which are associated with PTSD [60]. Moreover, the development of the DMN is particularly sensitive during childhood, with research suggesting its development could be affected by early and prolonged trauma [61, 62]. Given these factors are more strongly associated with CPTSD than PTSD [5], the finding that DSO symptom cluster emotion dysregulation was most related to EF suggests similar neurobiological mechanisms may be involved in CPTSD as those espoused for the overlap between EF and PTSD.

### Limitations

A number of limitations to the present study should be noted. Firstly, whilst the self-report measure of EF facilitated the collection of data from a larger sample, it has limited convergent validity with neuropsychological measures of EF [37]. However, as a self-report measure, the scale has strong psychometric properties [37] and self-report EF measures are strongly related to functional impairment [63]. Secondly, the scale does not include items measuring cognitive flexibility, although this would be difficult to capture on a self-report measure. Data were collected during the Covid-19 pandemic, and environmental factors related to restrictive measures at the time could have affected participants’ responses. However, our research suggests veterans’ mental health difficulties remained relatively stable throughout the pandemic. Finally, no causal relationships can be interpreted from the current findings due to the cross-sectional design of the study. However, the observed finding of an association between DSO symptom clusters and EF impairment builds on previous findings of similar association with PTSD clusters and this can inform future research and clinical studies.

### Implications for treatment

Taken together, the findings of the present study suggest that CPTSD interventions may – as observed with PTSD treatment outcomes [22] – result in better symptom improvement in patients who display greater inhibitory control in neuropsychological tests. By separately analysing both PTSD and DSO symptom clusters, the current study has highlighted the potential role of emotion dysregulation in the overlap between EF impairment and PTSD observed in previous studies [10–12]. Future research might explore whether veterans with better inhibitory control and working memory respond better to CPTSD interventions. For example, Enhanced Skills Training in Affective and Interpersonal Regulation (ESTAIR; [64]) is a modular CPTSD treatment which sequentially targets each DSO symptom – including emotion dysregulation. Future studies might explore whether building skills in emotion regulation reduces impairment in EF and subsequently improves recovery trajectories.

## Conclusions

This was the first study to explore the relationship between EF and CPTSD symptom clusters in a clinical sample of UK Armed Forces veterans. That DSO symptom clusters, in addition to PTSD clusters, were associated with EF builds on previous findings and suggests that CPTSD treatment outcomes could similarly be affected by levels of EF impairment in veteran patients. Future research should explore the clinical implications of these findings further.

## Data Availability

The datasets analysed during the current study are not publicly available due to patient confidentiality.

## Abbreviations

CPTSD: Complex posttraumatic stress disorder
DMN: Default mode network
DSO: Disturbances in self-organisation
EF: Executive function
PTSD: Posttraumatic stress disorder
